# Dynamic Optical Coherence Tomography of Blood Vessels in Cutaneous Melanoma—Correlation with Histology, Immunohistochemistry and Dermoscopy

**DOI:** 10.3390/cancers15174222

**Published:** 2023-08-23

**Authors:** Sandra Schuh, Elke Christina Sattler, Anna Rubeck, Stefan Schiele, Nathalie De Carvalho, Lotte Themstrup, Martina Ulrich, Gregor B. E. Jemec, Jon Holmes, Giovanni Pellacani, Julia Welzel

**Affiliations:** 1 Department of Dermatology, University Hospital Augsburg, 86179 Augsburg, Germany; julia.welzel@uk-augsburg.de; 2 Department of Dermatology and Allergy, University Hospital, Ludwig Maximilian University of Munich, 80337 Munich, Germany; elke.sattler@med.uni-muenchen.de; 3 Institute of Mathematics, University of Augsburg, 86159 Augsburg, Germany; anna.rubeck@math.uni-augsburg.de (A.R.); stefan.schiele@math.uni-augsburg.de (S.S.); 4 Department of Dermatology, University of Modena and Reggio Emilia, 41124 Modena, Italy; dranathaliedecarvalho@gmail.com (N.D.C.); giovanni.pellacani@gmail.com (G.P.); 5 Department of Dermatology, Roskilde Hospital, Health Science Faculty, University of Copenhagen, 4000 Roskilde, Denmark; lotte.themstrup@gmail.com (L.T.); gbj@regionsjaelland.dk (G.B.E.J.); 6 CMB Collegium Medicum Berlin, 10117 Berlin, Germany; m.ulrich@collegiummedicum.de; 7 Michelson Diagnostics, Maidstone ME14 5FY, UK; jon.holmes@vivosight.com

**Keywords:** melanoma, dynamic optical coherence tomography, dermoscopy, immunohistochemistry, blood vessels

## Abstract

**Simple Summary:**

The formation of new blood vessels is crucial for tumor progression and worsens the prognosis of melanoma patients. Now the prediction of risk progression depends on histologic parameters assessed after surgery. Dermoscopy also assesses pieces of information such as the vascularization of melanomas. Due to the possible in vivo evaluation of blood vessels in melanomas with dynamic optical coherence tomography (D-OCT), we wanted to examine atypical vessel patterns, density, shape and distribution as well as the presence/type of vessel branching in melanomas in three depths and to correlate the data with the same patterns with dermoscopic and histologic malignancy parameters (like ulceration, regression and inflammation) for the evaluation of risk for metastases. The aim is to show that tumor vasculature can be noninvasively assessed using D-OCT before surgery.

**Abstract:**

Dermoscopy adds important information to the assessment of cutaneous melanoma, but the risk of progression is predicted by histologic parameters and therefore requires surgery and histopathologic preparation. Neo-vascularization is crucial for tumor progression and worsens prognosis. The aim of this study was the in vivo evaluation of blood vessel patterns in melanoma with dynamic optical coherence tomography (D-OCT) and the correlation with dermoscopic and histologic malignancy parameters for the risk assessment of melanoma. In D-OCT vessel patterns, shape, distribution and presence/type of branching of 49 melanomas were evaluated in vivo at three depths and correlated with the same patterns in dermoscopy and with histologic parameters after excision. In D-OCT, blood vessel density and atypical shapes (coils and serpiginous vessels) increased with higher tumor stage. The histologic parameters ulceration and Hmb45- and Ki67-positivity increased, whereas regression, inflammation and PD-L1-positivity decreased with risk. CD31, VEGF and Podoplanin correlated with D-OCT vasculature findings. B-RAF mutation status had no influence. Due to pigment overlay and the summation effect, the vessel evaluation in dermoscopy and D-OCT did not correlate well. In summary, atypical vessel patterns in melanoma correlate with histologic parameters for risk for metastases. Tumor vasculature can be noninvasively assessed using D-OCT before surgery.

## 1. Introduction

Despite all public awareness, intervention and screening programs, the incidence of cutaneous melanoma is still rising fast, especially in Europe, North America and Australia. Thin melanomas are generally cured by simple surgery, while thicker melanomas with high-risk features tend to develop metastases to a great extent. So far, the predominant part of risk assessment for melanomas takes place ex vivo, i.e., after excision. The risk of progression in melanoma is determined by histologic parameters such as tumor thickness and ulceration, rate of mitosis and status of the sentinel lymph node [1]. All these can only be assessed after tumor excision ex vivo. Especially for neoadjuvant settings, noninvasive methods for diagnosing melanomas may be of importance. Among others, neo-vascularization is crucial for tumor progression and the development of metastases [2,3].

We, therefore, hypothesize that noninvasive imaging of melanoma vasculature may be a relevant parameter for in vivo risk assessment to supplement other diagnostic findings.

Vessel structures and patterns can be visualized by D-OCT in vivo noninvasively. Whereas characteristic vessel structures have already been well described for basal cell carcinoma by D-OCT [4,5], only pilot studies on the vessel structures in melanomas have been evaluated by D-OCT yet [5,6,7]. The objective of this paper is to explore the association between vascular patterns and shapes in D-OCT with dermoscopy and histologic features for risk assessment in melanoma.

## 2. Materials and Methods

The current study was part of the European Union-funded project ADVANCE “Automatic Detection of Vascular Networks for Cancer Evaluation” (Project number 621015).

The primary aim of the study was to describe correlations between specific microvascular patterns of melanomas with other risk factors and the development of metastasis. The study was conducted at four dermatology departments in Europe (Germany, Italy, and Denmark). All authors performed the study independently, in an unbiased manner, and based on all rules valid for clinical medicine. The study was approved by the respective institutional review boards and followed the principles according to the declaration of Helsinki at all times (for Augsburg the Ethical Committee of the Ludwig-Maximilian-University: Project number P30-14). Patients > 18 years with cutaneous melanomas were enrolled in the study after informed consent in a prospective way.

For the ADVANCE project, a total of 160 melanomas from 156 patients were evaluated. Among those, a subset of 49 histologically proven melanomas of 46 patients from the Augsburg center were eligible for evaluation in this study as they offered dermoscopy and histopathologic evaluation in addition to D-OCT examination. Lesions with clinical and/or specific dermoscopic features for melanoma were imaged with D-OCT before surgery and consecutive histopathology. All images were taken prospectively, stored, and visually evaluated afterward, but only melanomas with good-quality D-OCT and dermoscopy images as well as with confirmed histopathological diagnosis were included in the following study. Dermoscopy was performed using a contact and non-contact polarized and non-polarized light dermoscope, Dermlite DL200 HR Hybrid, with a 10-fold magnification (3Gen, S. Juan Capistrano, CA, USA), and the non-polarized contact dermoscope camera VivaCam^®^ (in Vivascope 1500, MAVIG GmbH, Munich, Bavaria, Germany). The OCT image-taking procedure was performed as described in previous studies [8].

The D-OCT images were taken with the D-OCT device VivoSight^®^ from Michelson Diagnostics (Maidstone, Kent, UK), which was developed further during the project and received CE certification. The device offers an axial resolution of <10 µm and a lateral resolution of <7.5 µm. Moreover, the probe of the OCT device carries a color camera, which allows the exact positioning of the probe on the lesion. For the preparation of the skin, there is no need for gel or oil. The center of the tumor and the healthy adjacent or opposite skin were measured as an area of 6 mm × 6 mm and 1–2 mm in depth. The D-OCT works well until 0.5 mm; deeper into the skin, the image quality becomes blurry due to background noise. The whole imaging process takes 30 s and produces 120 vertical images with a space of 0.05 mm in between, creating a 3D block of 6 mm × 6 mm × 2 mm (width/length/depth) with an image pixel size of 4.3 µm. The smallest detectable vessel size is 20 µm. The en-face D-OCT images are horizontal slices created automatically by the software from the top in the fitted en-face mode and can be shown at any desired depth below the surface. The horizontal picture contains the structural information together with the red pixels, which represent the moving particles from the blood flow of the vessels at a certain depth. Small changes in blood flow can be detected within a very short time using a method called speckle variance [9]. In contrast, very slowly flowing or stationary blood cannot be detected. Therefore, the intensity of the red overlay is a marker for the power of the D-OCT signal.

D-OCT measurements were performed in the center of the lesion and, if possible, on adjacent healthy skin. Afterward, the vessel morphologies were reviewed in both levels (mainly horizontal, but also vertical). For a microvascular evaluation of the vessels in each melanoma scan, the OCT-Fitter V2.1 (created by Marco Manfredi and Constantino Grana, Modena) was used at three standardized depths: 150, 300 and 500 µm [10]. For a standardized assessment, the previously described terminology by Ulrich et al. was applied on the D-OCT images [8,11]. At the three depths, the vessel density, diameter, distribution, orientation, pattern, branching and direction were analyzed. As a reference for the vessel density and diameter in melanomas, blood vessels in healthy skin were used. Furthermore, the vessel morphology in the shape of “dots”, “blobs”, “coils”, “lines”, “curves” and “serpiginous forms” was recorded as present or absent. One type of vessel shape was noted as present if it could be seen in a single horizontal D-OCT image at least three times. 

The vertical and horizontal D-OCT images were assessed by three dermatologists (SS, NDC, JW), who are very experienced in D-OCT image evaluation, and the dermoscopic images were reviewed by one dermatologist (ES), who is an expert in this field. The dermoscopic images were evaluated in relation to visible or non-visible vessels due to pigment overlay in % [12]. The histology and immunohistochemistry slides were assessed by one board-certified dermatopathologist (JW). The dermatologists were all blinded to the respective other technique (either D-OCT or dermoscopy or histopathology), the Breslow thickness and clinical information. D-OCT image assessment was conducted blindly by SS and NDC on two different laptops. If there was a disagreement, the third expert (JW) was consulted to find a consistent decision. 

**Figure 1:**
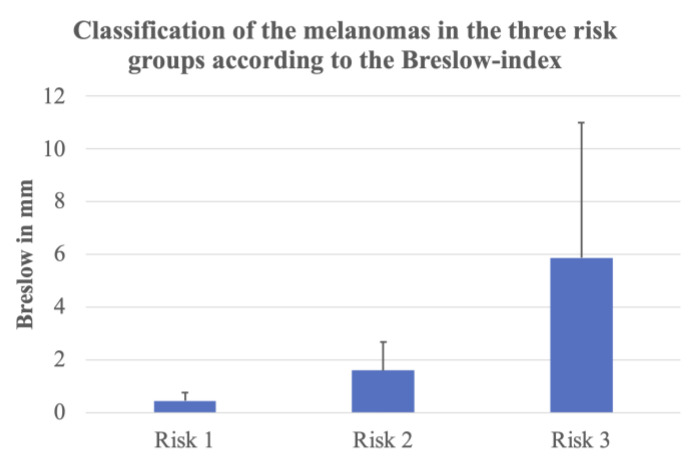
Classification of the 49 melanomas in the three risk groups according to the Breslow index: R1 = low risk, R2 = intermediate risk, R3 = high risk. The bars show the mean tumor thickness in each risk group in mm. Risk group 1 (low risk): in situ melanoma and stage IA (thickness **<** 1 mm without ulceration). Risk group 2 (intermediate risk): stage IB to stage IIB (intermediate thickness with or without ulceration). Risk group 3 (high risk): stage IIC (ulcerated melanoma > 4 mm), stage III (lymph node involvement) and stage IV (distant organ metastases).

Post-operative histopathological results, the status of the sentinel lymph node (when performed) and the primary staging according to AJCC 8th edition [13] led to the formation of three groups (Figure 1) [14,15].

All 49 melanoma slides were reviewed for ulceration, regression and inflammation (all 0 = not present, 1 = a little present, 2 = present, 3 = a lot present) and were immunohistochemically stained for Hmb45 (0 = negative, 1 = epidermis, 2 = upper dermis, 3 = lower dermis), CD31 and Podoplanin (0 = not present, 1 = a little present, 2 = present, 3 = a lot present), VEGF (0–3), BRAF (0 = negative, 1 = positive), PD-L1 (0 = negative, 1 = <5%, 2 = 5–50%, 3 = >50%) and Ki67 (dermis, 0 = negative, 1 = <5%, 2 = 5–50%, 3 = >50%).

The data were collected with Microsoft^®^ Excel^®^ for Mac Version 16.75.2, and statistical evaluations were performed with IBM^®^ SPSS^®^ Statistics Software for Mac (SPSS 24.0, IBM Corp., Armonk, NY, USA). Statistical analysis for comparison between the risk groups concerning ulceration, regression, inflammation, BRAF, Hmb45, Ki67, Podoplanin, VEGF, CD31, PD-L1, blood vessel density and shape was performed using the Mann–Whitney U-test. A *p*-value below 0.05 was considered as statistically significant. For the correlation between D-OCT, dermoscopy and histology, phi coefficients were calculated.

## 3. Results

### 3.1. Cutaneous Melanomas

The study analyzes a subset of 49 melanomas from 46 patients (13 females, 33 males) from a D-OCT prospective multicenter melanoma study [7] of 160 melanomas, for which the histological slides and all immunohistochemistry stains as well as dermoscopic images were available for evaluation. Four melanomas had to be excluded from the dermoscopic assessment due to poor image quality or because there were no images available. Therefore, 49 melanomas fulfilled the inclusion criteria for the correlation of D-OCT with the histological and immunohistochemical findings, and 45 D-OCT images of melanomas could be correlated with dermoscopy. The patients’ age in this subgroup of 49 melanomas ranged from 29 to 95 years (mean age 69 years). After the imaging procedure, all patients were treated with the standard removal of the melanoma according to German melanoma guidelines [16] with a safety margin according to the tumor thickness, and if indicated, sentinel lymph node biopsy and staging were performed. The stage was classified using the Eighth American Joint Committee on Cancer (AJCC) melanoma classification [17].

Melanomas were located on the head and neck (11/49; 22.5%), trunk (30/49; 61.2%) and extremities (8/49; 16.3%). Melanomas were classified as follows: 21 (42.9%) superficial spreading melanomas, 13 (26.5%) nodular melanomas, 1 (2.0%) acrolentiginous and 3 (6.1%) lentigo maligna lesions, and 4 (8.2%) invasive lentigo maligna melanomas. Seven (14.3%) melanomas could not be classified because of mixed features. Twelve of them (24.5%) showed ulceration. Five (10.2%) melanomas were in situ melanomas, and 23 (46.9%) invasive ones were up to 1 mm thick. Eleven (22.5%) melanomas were between 1 and 2 mm, three (6.1%) were between 2 and 4 mm and seven (14.3%) melanomas were thicker than 4 mm.

After excision, sentinel lymph node biopsy and staging, 23 (46.9%) melanomas were classified as risk group I (in situ to stage IA); 15 (30.6%) melanomas were classified as risk group II (stage IB to stage IIB); and 11 (22.5%) melanomas were classified as risk group III, including stage IIC with a thick ulcerated tumor (3/11 cases; 27.3%), stage III with lymph node involvement (6/11 cases; 54.5%) and stage IV with distant organ metastases (2/11 cases; 18.2%). Of the 11 melanomas with metastasis, 3 (3/11; 27.3%) melanomas were 1 to 2 mm thick, 2 melanomas (2/11; 18.2%) were 2 to 4 mm thick and 6 (56/11; 4.5%) melanomas were more than 4 mm thick. Seven of these cases (7/11; 63.6%) showed a purely nodular subtype; five (5/11; 45.5%) presented ulceration.

### 3.2. Blood Vessels in D-OCT and Comparison with Histology

All melanomas showed distinct vessel patterns in the horizontal D-OCT images at the three depths 150 µm, 300 µm and 500 µm. In the following, the results are presented at 300 µm, which is the most effective depth for the evaluation of the vessel patterns as other studies confirmed [7,18,19]. The most common vessel patterns in D-OCT were dots. Coiled and serpiginous vessels increased with risk group at 300 µm. The correlations between dermoscopy, D-OCT and histology for the most important vessel types (like dots, coils and serpiginous vessels [7]) in cutaneous melanomas were investigated. The correlations for coils were very weak between D-OCT and histology (j = 0.175), between D-OCT and dermoscopy (j = 0.055) and between histology and dermoscopy (j = 0.081). For serpiginous vessels, the correlations were also weak between histology and D-OCT (j = 0.206), between D-OCT and dermoscopy (j = 0.263) and between histology and dermoscopy (j = 0.06). The vessel pattern dots showed a negative correlation between histology and dermoscopy (j = −0.201), showing that even if dots are the predominant vascular pattern in histology, they are not recognized as such in dermoscopy. There was a weak correlation between D-OCT and histology (j = 0.049), as well as between D-OCT and dermoscopy (j = 0.086). In contrast to D-OCT, where the vasculature was assessed in horizontal images, histology provides cross-sectional slides. The procedure of excision, fixation and slicing of the tissue leads to a collapse of the blood vessels. Therefore, it is much more difficult to assess vessel patterns, networks and branching in histologic sections.

**Figure 2:**
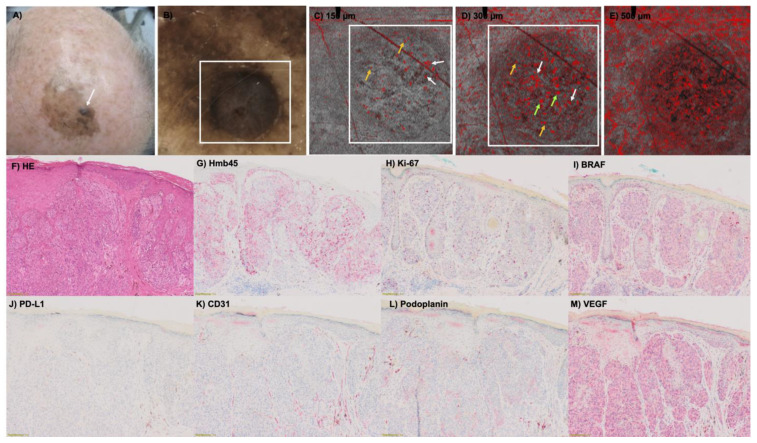
(**A**) Clinical image of a pigmented lesion located on the scalp with the presence of a nodular component (white arrow). The case corresponds to a secondary nodular cutaneous melanoma on the head, tumor thickness 1.1 mm, R2. (**B**) Dermoscopy of the nodular area (white arrow) displaying intense hyperpigmentation obscuring the visualization of vascular structures (in ×10 magnification). (**C**,**D**) D-OCT images (6 × 6 mm^2^) captured in the nodular area (white square). (**C**) At 150 µm depth, dotted vessels are predominant (yellow arrows) but curved-shape vessels are also seen (white arrows). (**D**) At the depth of 300 µm, an intense pleomorphism of vascular morphologies is detected, with the presence of dotted (yellow arrows), blob (green arrows) and curved (white arrows) vessels like in (**E**) at 500 µm. Histological stainings (in ×4 magnification) of the melanoma in HE (**F**), Hmb45 (**G**), Ki-67 (**H**), BRAF (**I**), PD-L1 (**J**), CD31 (**K**), Podoplanin (**L**) and VEGF (**M**). Hm45, Ki-67, CD31, Podoplanin and VEGF increase with risk, PD-L1 decreases with risk and the BRAF status has no influence.

**Figure 3:**
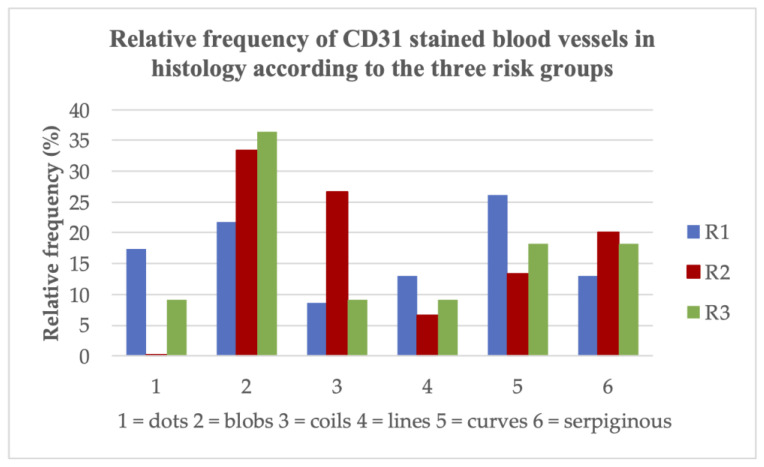
Relative frequency of CD31-stained blood vessels in histology according to the three risk groups. R1 = low risk, R2 = intermediate risk, R3 = high risk.

**Figure 4:**
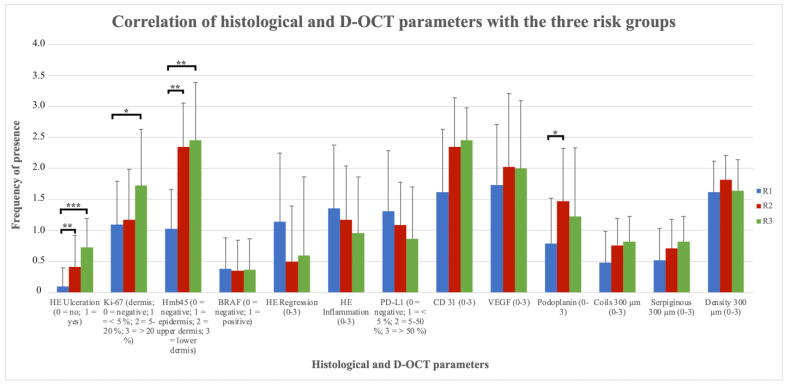
Correlation of histological and D-OCT parameters with the three risk groups. Vessel shapes (coils and serpiginous) at 300 µm; vessel density; histological vessel parameters like CD31, VEGF and Podoplanin; regression; inflammation; PD-L1-positivity; and the histological parameters for malignancy like ulceration, Hmb45 (activation), Ki-67 (proliferation) and BRAF status in the histological slides of the melanomas correlated with the three risk groups R1-R3. Remarks: HE = hematoxylin eosin staining; R1 = risk group 1, R2 = risk group 2, R3 = risk group 3 according to the Breslow index; * = statistical significance (*p*-value below 0.05); ** = statistical significance (*p*-value below 0.001); *** = statistical significance (*p*-value below 0.0001).

**Figure 5:**
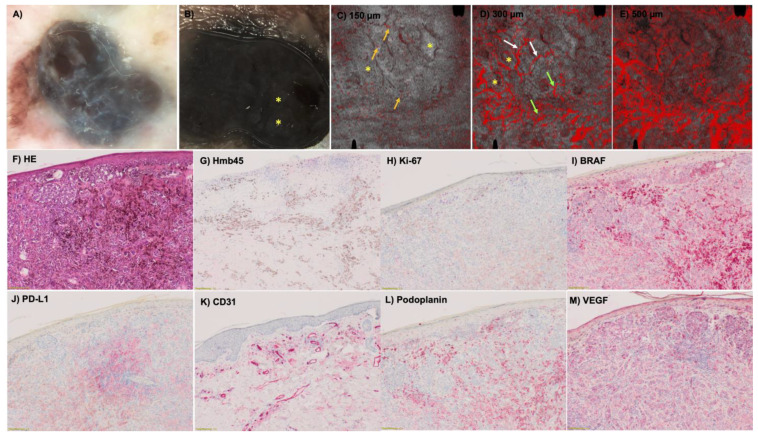
Dermoscopic (**A**,**B**) and D-OCT images (6 × 6 mm^2^) at 150 (**C**), 300 (**D**) and 500 µm (**E**) of a nodular melanoma. The case corresponds to a nodular cutaneous melanoma on the left shoulder, tumor thickness 3.35 mm, R3. (**B**) Dermoscopy of the nodular component displaying intense hyperpigmentation obscuring the visualization of vascular structures (in ×10 magnification). (**C**,**D**) D-OCT images captured in the nodular area. (**C**) At the depth of 150 µm, dotted vessels are seen (yellow arrows) within an irregular structural surface limited by dark slits giving rise to structural islands (yellow asterisks), which is also identified in dermoscopy (yellow asterisks in (**B**)). (**D**) At the depth of 300 µm, serpiginous vessels are predominant (white arrows), with some displaying arborizing branches (green arrows). We note some serpiginous vessels are organized around the structural islands (yellow asterisks) occupying the dark slits described in (**C**), suggesting an intense neoangiogenesis to nourish deep nests of atypical melanocytes. Mainly serpiginous vessels can be seen in D-OCT. Histological stainings (in ×4 magnification) of the melanoma in HE (**F**), Hmb45 (**G**), Ki-67 (**H**), BRAF (**I**), PD-L1 (**J**), CD31 (**K**), Podoplanin (**L**) and VEGF (**M**). Hm45, Ki-67, CD31, Podoplanin and VEGF increase with risk, PD-L1 decreases with risk and the BRAF status has no influence.

It has already been shown that different vessel types in D-OCT could be found in melanomas according to the three risk groups [7,19]. But is it also possible to prove these findings in histology? The histological examination and measurement of the Breslow index allowed the classification of the 49 melanomas in this study into three risk groups (described above): R1 = low risk, R2 = intermediate risk, R3 = high risk (Figure 1). The vessel criteria in the histologic slides were assessed at a depth of 300 µm. In in situ and very thin melanomas, the vessels are located below the tumor, whereas in thicker melanomas, the vessels are located inside the tumor. In lower risk groups like R1 and R2, mainly dots, blobs and sometimes curved vessels could be detected in D-OCT (see for an example Figure 2. This also correlated with the histology results, where these vessels could be visualized with the CD31 staining (Figure 3). Coils and serpiginous vessels are mainly and sometimes only found in D-OCT in melanomas of the high-risk group R3 (Figure 4, see for an example Figure 5). The other vessel shapes were not as common in the high-risk group R3. This fact correlates also very well with the CD31-stained vessels in histopathology (Figure 3 and Figure 4).

The well-known histologic parameters for malignancy like ulceration, Hmb45 (activation) and Ki-67 (proliferation) were associated with the above-mentioned risk groups R1-R3. It could be confirmed that the named histologic parameters increased with risk (Figure 4). The ulceration status was significantly different between R1 and R2 (*p* = 0.008) and showed a highly significant difference between R1 and R3 (*p* < 0.001), but not between R2 and R3 (*p* = 0.19). For Hmb45, a significant difference was found between R1 and R2 (p = 0.001) and between R1 and R3 (*p* = 0.001), but not between R2 and R3 (*p* = 0.30). Ki-67 differed significantly between R1 and R3 (*p* = 0.020), but not between R1 and R2 (*p* = 0.21) and R2 and R3 (*p* = 0.24). The B-RAF mutation status had no association with risk (Figure 4). The three groups did not show a significant difference in relation to the BRAF status (R1–R2: *p* = 0.72; R2–R3: *p* = 0.88; R1–R3: *p* = 0.88). In contrast, the degree of regression, inflammation and PD-L1-positivity tended to be associated with lower risk as a sign of immune response (Figure 4). The difference for regression (R1–R2: *p* = 0.64; R2–R3: *p* = 0.41; R1–R3: p = 0.26), inflammation (R1–R2: *p* = 0.10; R2–R3: p = 0.92; R1–R3: *p* = 0.15) and PD-L1-positivity (R1–R2: *p* = 0.46; R2–R3: *p* = 0.47; R1–R3: *p* = 0.21) between the three risk groups did not reach significance. Blood vessel density, the presence of certain vessel shapes (like coils and serpiginous vessels) and the VEGF status showed a tendency toward higher values for higher risk classes (Figure 4). However, the differences regarding blood vessel density (R1–R2: *p* = 0.33; R2–R3: *p* = 0.36; R1–R3: *p* = 0.93), vessel shapes (coils R1–R2: *p* = 0.20, R2–R3: *p* = 0.62, R1–R3: p = 0.10; serpiginous vessels R1–R2: *p* = 0.96, R2–R3: p = 0.24, R1–R3: *p* = 0.23; lines R1–R2: *p* = 0.84, R2–R3: *p* = 0.91, R1–R3: *p* = 0.96; curves R1–R2: *p* = 0.08, R2–R3: *p* = 0.06, R1–R3: *p* = 0.73) and VEGF status (R1–R2: *p* = 0.09; R2–R3: *p* = 0.70; R1–R3: *p* = 0.26) did not reach significance between the three risk groups in the histology sections. For the vessel shape dots, it did not make sense to perform these tests, since only one patient did not have dots. Only the number of blobs compared between risk groups 2 and 3 was statistically significant (R1–R2: *p* = 0.06; R2–R3: *p* = 0.03; R1–R3: *p* = 0.73). Nevertheless, there was no significant difference between the three risk groups for the histological vessel parameter CD31 (R1–R2: *p* = 0.12; R2–R3: *p* = 0.65; R1–R3: *p* = 0.058). For Podoplanin staining, a significant difference could be found between R1 and R2 (*p* = 0.007), but not for R2 and R3 (*p* = 0.29) and not for R1 and R3 (*p* = 0.33).

### 3.3. Comparison between Dermoscopy and D-OCT

Dermoscopy provides an image of the skin surface. Depending on parameters like the thickness of the stratum corneum and the epidermis as well as pigmentation, the skin is more or less translucent. Therefore, only structures of superficial parts of the skin down to about 200 µm are visible, which is a general limitation of the analysis of deeper vessels. The difference between cross-polarized and non-polarized dermoscopy is that cross-polarized dermoscopy does not require direct skin contact. Therefore, blood vessels can be seen more prominently due to a lack of pressure [20,21,22]. Furthermore, blood vessels in deeper skin levels are more evident with polarized dermoscopy. Since we used a hybrid dermoscope, complementary information of both techniques was assessed, but most of the hybrid devices and also the Vivacam^®^ are used in contact with skin, leading to possible exsanguination of the vessels and making it harder to diagnose skin lesions [20,21,22].

**Figure 6:**
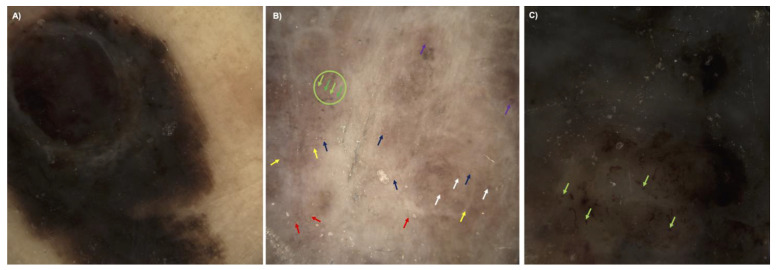
Dermoscopy images (×10 magnification), in which the vessel criteria of D-OCT images cannot be easily applied, on their own are not useful for the risk assessment of melanoma. (**A**) Dermoscopy of a nodular cutaneous melanoma on the left calf, tumor thickness 1.7 mm. Vessels are not visible due to the hyperpigmentation overlay. (**B**) Superficial spreading cutaneous melanoma on the back, tumor thickness 1.2 mm with regression zone. Only in the regression zone, discrete vessels are visible in the form of dots (green arrows inside green circle), blobs (white arrows), lines (yellow arrows), curves (dark blue arrows), coils (purple arrows) and serpiginous vessels (red arrows). (**C**) In this nodular cutaneous melanoma on the back, tumor thickness 5.3 mm, the discrimination between pigmented (darker green arrows) and vascular dots (light green arrows) impedes the image evaluation.

**Figure 7:**
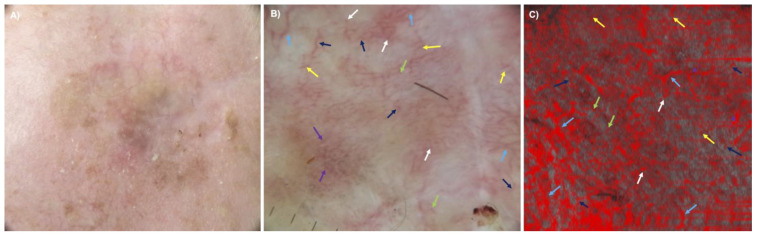
Clinical (**A**), dermoscopic (**B**, ×10 magnification) and D-OCT (**C**) images (at 300 µm depth, 6 x 6 mm^2^) of a hypopigmented in situ melanoma on the forehead. Vessels can be clearly seen in dermoscopy (**B**) and in the D-OCT image at 300 µm (**C**). Blood vessels are visible in the form of dots (green arrows), blobs (white arrows), lines (yellow arrows), curves (dark blue arrows), coils (purple arrows) and serpiginous vessels (light blue arrows) in dermoscopy (**B**) and D-OCT (**C**) images.

The descriptive comparison between the two methods regarding vascular pattern analysis contained some problems. In dermoscopy, the vessels were not visible due to the pigment overlay (Figure 6A,C), or the discrimination between pigmented dots and vascular dots impeded the evaluation of the images (Figure 6C). When vessels were visible, the most common vessel types were dots (11/45), blobs (2/45), coils (4/45), lines (28/45), curves (26/45) and serpiginous vessels (5/45) in dermoscopy. More details on vessel types and distribution in dermoscopy can be found in the table in the supplement (Table S1). Figure 7 shows a hypopigmented melanoma, which depicts blood vessels in dermoscopy and D-OCT. In addition, mostly dermoscopic pictures showed a pigment overlay comprising 60–100% of the lesion (see Figure 6A) or pigmentation only inside a regression area—both hampering the visualization of very discrete vessels (Figure 6B). In these cases, vessels in dermoscopy corresponded to coils, curves, lines and dots in D-OCT. These are the reasons why the vessel criteria of D-OCT images evaluated in a depth of 300 µm cannot be easily applied or transferred to dermoscopic pictures of visible vessels at the surface. In strongly pigmented melanomas, deeper vessels at a depth of 300 µm are rarely visible in dermoscopy, whereas in D-OCT, the pigmentation does not affect the visibility of blood vessels. Therefore, the evaluation of blood vessels in dermoscopy itself is helpful for the diagnosis of melanoma vs. benign melanocytic lesions but not useful for the risk assessment of melanoma. Nevertheless, if present, the typical dermoscopic criteria for melanoma blood vessels are certainly identifiable.

## 4. Discussion

In summary, OCT is well established for the diagnosis of non-melanoma skin cancer (NMSC) [2,23] but so far has not been proven to be useful in the diagnosis of melanocytic tumors [24,25,26,27,28]. With the newer development of D-OCT, the option of studying vascular parameters has become available, also providing new insights into melanocytic lesions [6,7,19]. Therefore, in this preliminary study, we explored the hypothesis that vessel shape and distribution are associated with known markers of tumor progression in melanoma. Our data suggest that blood vessel density and atypical shapes like coils and serpiginous vessels increased with higher tumor stages, and D-OCT may prove to have a role as an additional parameter for estimating the risk of metastasis before excision. This may be of special interest when treating metastatic melanomas in a neo-adjuvant setting where histology of the whole tumor is still lacking. As expected, the histologic parameters ulceration and Hmb45- and Ki67-positivity increased with risk, whereas the degree of regression, inflammation and PD-L1-positivity correlated with lower risk. The BRAF mutation status showed no significant influence. The histologic vessel parameters (CD31, VEGF and Podoplanin) correlated well with the findings of D-OCT. Due to the summation effect and the pigment overlay, vessel patterns in dermoscopy hardly correlated with D-OCT results.

In accordance with pilot studies on melanocytic and non-melanocytic lesions using D-OCT [6,18,19], the best depth to identify vessel patterns was confirmed to be at 300 µm. Using the nomenclature suggested by Ulrich et al. [8], coils and serpiginous vessels were seen in correlation with higher tumor stage and greater risk of progression, as previously demonstrated by De Carvalho et al. [7,19].

### Limitations

In dermoscopy, certain vessel patterns like irregular vessels with different calibers, hair needle vessels and the combination of many different patterns give important clues in the diagnosis of melanoma and are well described in the literature [12,29], especially in amelanotic melanoma [30]. Yet, in our images, the majority of lesions showed few vessels and a very high percentage of overlay due to pigment. Therefore, in this subset of data also considering the vessel patterns seen, there was no consecutive correlation with the vascular patterns described in D-OCT. Still, the diagnosis of melanoma could be sustained by typical dermoscopic features which are well established and described in detail elsewhere [31,32,33]. It must be kept in mind that in dermoscopy, only the skin surface is visible, with little information from deeper parts of the skin. The dermoscopic 2D pictures sum up the information from superficial layers of the skin, while in D-OCT, 3D images of the skin allow the visualization of tissue down to 1.5 mm [34].

One limitation of D-OCT is an intermediate resolution—single-cell depiction comparable to histopathology or confocal microscopy is not possible.

## 5. Conclusions

In conclusion, our work indicates that D-OCT visualizes tumor vessels in melanoma. Since atypical blood vessel patterns in D-OCT correlate with greater tumor thickness, D-OCT can provide additional information about a risk constellation [7,19]. The correlation of D-OCT with dermoscopy was not high, probably due to heavily pigmented tumors masking vessel patterns.

## Data Availability

Pseudonymized data are available on request.

## Supplementary Materials

The following supporting information can be downloaded at: https://www.mdpi.com/article/10.3390/cancers15174222/s1, Table S1: Evaluation of the number of vessels, vessel types and distributions of the melanomas in dermoscopy.
