# Supplementary material for: Dynamic Optical Coherence Tomography of Blood Vessels in Cutaneous Melanoma—Correlation with Histology, Immunohistochemistry and Dermoscopy
Source: Cancers (Basel). 2023 Aug 23;15(17):4222. doi: 10.3390/cancers15174222 (PMC10487152; doi:10.3390/cancers15174222)
Supplement: Supplementary file 1 [file cancers-15-04222-s001.zip › cancers-2516526-supplementary.pdf]

CASE ID Lesion	Metastasis	Risk group	DOT 300µm	DOT DISTRIBUTION 300µm	BLOB 300µm	BLOB DISTRIBUTION 300µm	COILED 300µm	COILED DISTRIBUTION 300µm	LINE 300µm	LINE DISTRIBUTION 300µm	CURVED 300µm	CURVED DISTRIBUTION 300µm	SERPIGINOUS 300µm	SERPIGINOUS DISTRIBUTION 300µm	PATTERN 300µm	BRANCHING 300µm	DENSITY 300µm	DIAMETER 300µm	ORIENTATION 300µm	CROSS-SECT. COLUMNS	CROSS-SECT. SPIKES
20	0	2	1	2	1	2	0	0	0	0	1	2	1	2	1	0	2	2	0	0	1
29	0	1	1	3	0	0	0	0	0	0	0	0	0	0	0	0	1	1	0	0	0
30	0	1	1	3	0	0	0	0	1	2	1	2	1	2	2	1	2	2	0	1	1
34	0	2	1	1	1	1	1	1	0	0	1	1	1	1	1	0	1	1	2	1	1
35	0	1	1	3	1	3	1	2	1	3	1	2	1	2	2	1	2	2	1	1	1
38	0	2	1	2	1	2	0	0	0	0	0	0	1	3	1	0	2	2	0	1	1
40	0	1	1	2	0	0	0	0	0	0	1	3	1	3	1	0	1	1	0	1	1
41	0	1	1	2	0	0	0	0	0	0	1	2	1	2	1	0	1	1	0	0	1
42	0	1	1	2	1	2	1	2	0	0	1	2	1	2	2	1	2	2	0	1	1
43	0	2	1	2	1	2	1	2	0	0	1	2	0	0	1	0	1	1	0	1	1
44	0	2	1	2	1	2	0	0	0	0	1	2	0	0	1	0	2	2	0	1	1
51	0	1	1	2	1	2	1	2	1	1	1	2	1	2	2	1	2	2	0	1	1
57	1	3	1	2	1	3	1	2	0	0	1	2	1	2	1	0	1	1	0	1	1
66b	0	1	1	1	1	2	0	0	0	0	0	0	0	0	1	0	1	1	0	0	1
66c	0	1	1	2	1	2	1	2	1	1	1	2	0	0	1	0	1	1	0	0	0
66f	0	1	1	2	1	2	1	2	0	0	1	2	1	2	1	0	1	1	0	1	1
69	0	1	1	2	0	0	0	0	0	0	1	2	0	0	1	0	1	1	0	1	1
85	0	2	1	2	1	2	0	0	1	2	1	2	1	2	1	0	2	1	0	1	1
86	0	2	1	2	1	2	1	2	1	3	1	2	1	2	1	0	1	1	0	1	1
87s	0	1	1	2	1	2	0	0	0	0	0	0	0	0	1	0	2	1	0	1	1
87b	0	1	1	2	1	2	0	0	0	0	0	0	0	0	1	0	1	1	0	1	1
88	1	3	1	2	1	2	1	2	0	0	1	2	1	2	1	0	2	1	0	1	1
89	0	1	1	2	1	2	1	2	0	0	0	0	0	0	1	0	2	1	0	1	1
98	0	1	1	2	1	2	1	2	0	0	0	0	1	2	2	1	2	2	0	1	1
101	0	3	1	2	1	2	1	2	0	0	1	2	1	2	1	0	1	1	0	1	1
102	0	1	1	1	1	2	1	2	0	0	1	2	1	2	2	1	2	1	0	1	1
103	1	3	1	2	1	2	1	2	0	0	0	0	0	0	1	0	2	1	0	0	1
104	1	3	1	2	0	0	1	2	1	1	0	0	1	2	2	1	2	2	0	1	1
105	1	3	1	2	0	0	0	0	0	0	0	0	0	0	1	0	1	1	0	0	0
106	0	1	1	2	1	2	1	2	0	0	1	2	1	2	2	1	2	2	1	1	1
107	0	2	1	2	1	3	1	2	0	0	1	2	1	2	1	0	2	1	0	1	1
108	0	2	1	2	1	2	1	2	0	0	1	2	0	0	1	0	2	1	0	1	1
109	1	3	1	2	1	2	1	2	0	0	1	2	1	2	1	0	2	2	0	1	1
110	0	2	1	2	1	2	1	2	0	0	1	2	0	0	1	0	2	1	0	1	1
112	0	3	1	2	1	2	1	2	0	0	1	2	1	2	1	0	2	1	0	1	1
113	0	2	1	2	1	2	1	2	0	0	1	2	1	2	1	0	2	1	0	1	1
115	0	1	1	2	1	2	1	2	0	0	1	2	1	3	1	0	2	1	0	0	0
116	0	1	1	2	1	2	0	0	0	0	1	2	0	0	1	0	2	1	0	1	1
118	1	3	1	2	1	2	1	2	1	3	1	2	1	2	2	1	2	1	0	1	1
120	1	3	1	2	1	2	1	2	0	0	0	0	1	2	1	0	1	1	0	0	1
121	0	1	1	1	1	1	1	2	0	0	1	2	1	3	1	0	2	1	0	1	1
122	0	1	1	2	1	2	1	2	0	0	1	2	1	2	1	0	2	1	0	1	1
123	0	2	1	2	1	2	1	2	0	0	1	2	0	0	1	0	2	1	0	0	1
124	0	1	1	1	1	1	0	0	0	0	0	0	0	0	1	0	2	1	0	1	1
125	0	1	1	2	1	2	0	0	0	0	1	2	1	2	1	0	2	1	0	1	1
126	0	2	1	2	1	2	1	2	0	0	1	2	1	2	1	0	2	2	0	1	1
128	0	3	0	0	0	0	0	0	0	0	1	2	1	2	1	2	2	2	1	1	1
129	0	2	1	3	1	2	1	3	1	2	1	2	1	2	1	2	2	2	0	1	1
130	0	2	1	2	1	2	1	2	0	0	1	2	0	0	1	0	2	1	0	0	1
